# *Plasmodium falciparum* malaria in northern Côte d’Ivoire: prevalence in the general hospital of Tanda sanitary district

**DOI:** 10.5281/zenodo.10784997

**Published:** 2016-01-07

**Authors:** Thomas Y. Aba, Raoul Moh, Lassina Cissé, Gisele C. Yapo-Kouadio, Frederic N. Ello, Chrysostome Mossou, Zelica Diallo, Ouffoue Kra, Emmanuel Bissagnené

**Affiliations:** 1 Département de Santé Publique et Infectiologie, Université Alassane Ouattara de Bouaké, Bouaké, Côte d’Ivoire; 2 Service des Maladies Infectieuses et Tropicales, CHU de Treichville, Abidjan, Côte d’Ivoire; 3 Service de Pédiatrie, CHU de Treichville, Abidjan, Côte d’Ivoire; 4 Service de Parasitologie, CHU de Cocody, Abidjan, Côte d’Ivoire

## Abstract

**Background:**

Until about 2010, the majority of data collected on malaria in Côte d’Ivoire were based on presumptive cases, particularly in the northern part of the country, where parasitological research had rarely been carried out. Recently, WHO recommended restricting treatment to confirmed malaria cases only. Thus, the purpose of this study determine the actual malaria prevalence amongst presumptive cases admitted to one of the general hospitals in the Northern part of the country, where malaria diagnosis is suboptimal.

**Materials and methods:**

A cr oss-sectional study was conducted in the general medicine, maternity and paediatric wards between January and August 2010. Patients of all ages, suspected of having malaria, were included after giving their informed oral consent. Several parameters were investigated: the presence of *Plasmodium* using thick blood film, HIV/ *Plasmodium* co-infection, signs of severity, aspects of malaria treatment and other associated factors.

**Results:**

Of 379 patients included, with a median age of 4 yrs [range 1 month - 71 yrs], 9% were HIV-positive, 74% were ≤ 15 yrs of age, 60% were urbanised and 23% were using long-lasting insecticide-treated nets. Malaria prevalence was 67.5% and was significantly associated with the rainy season (p < 0.001), age ≤ 5 yrs (p = 0.004) and no cotrimoxazole chemoprophylaxis in HIV-infected patients (p = 0.04). Only *P. falciparum* was detected, with a mean density of 12,523 trophozoites/μl of blood, but with 12,610 trophozoites/μl of blood in HIV-positive patients and 7,055 trophozoites/μl of blood in HIV-negative patients (p < 0.001). Severe malaria accounted for 77% of cases. Prescribed antimalarial drugs were: IM artemether (56%), quinine (28%), artemether + lumefantrine (10%) and artesunate + amodiaquine (6%). Apyrexia and parasite clearance were observed at day 2-3 post treatment in 87% of patients. Adverse events were reported among 60 patients (17%). The outcome was marked by: a healing rate of 90%, a rate of 5% lost to follow-up and a 7% lethality for severe malaria, significantly associated with the age ≤ 5 yrs (p=0.02), hyperparasitaemia >20% (p=0.004), neurological disorders (p < 0.001) and respiratory distress (p=0.007).

**Conclusions:**

Malaria prevalence in the general hospital of Tanda remains high, with a predominance of sever e malaria affecting children under the age of 5 yrs.

## 1 Introduction

Currently there is renewed interest in the fight against malaria with support from many initiatives, which raises the possibility of its elimination or global eradication. In order to monitor progress it is essential that the actual malaria prevalence in a given region or country should be known. Nevertheless, in many parts of Africa such data remain absent, which is largely also the case in Côte d’Ivoire where much diagnosis has been based on presumptive cases with fever as the major clinical manifestation [1]. Fever has historically been the most commonly used characteristic for diagnosing malaria, both in health centres and at home [2]. However, many experts believe that in 40 -50% of the cases diagnosed and treated with antimalarial drugs, based solely on signs of febrile illness, these are not malaria cases [3-5]. Some recent studies in urban sub-Saharan Africa even reported rates of misdiagnosis of 90% and above [6]. At present, the World Health Organization (WHO) recommends limiting the use of artemisinin-based combination therapies (ACTs) only to parasitologically-confirmed cases of malaria [7]. However, in Côte d’Ivoire, there are few available data on the morbidity and mortality of actual malaria cases in the hospitals far away from Abidjan, the economic capital in the south [8]. The aim of this study was to provide data on the confirmed malaria cases in a referral hospital in northern Côte d’Ivoire, the General hospital of Tanda sanitary district, where malaria diagnosis was still suboptimal.

## 2 Materials and methods

### 2.1 Setting and study site

Tanda district is located in the northeast of Côte d’Ivoire in the Zanzan region, 395 km from Abidjan. The ecosystem consists of moist wooded savannah. The region has a long (April to mid-July) and a short rainy season (mid-July to September), then a short (mid-September to November) and a long dry season (December to March). It is fed by the Comoé River and the Baya and N’djoré watercourses. Tanda sanitary district has one general hospital, three urban health centres and 23 rural health centres. The district has 173,173 inhabitants [9], residing in one prefecture (Tanda city), five sub-prefectures and five rural districts, subdivided into 144 villages. Data were collected in the general medicine, maternity and paediatrics wards of the general hospital of Tanda (GHT). GHT has a centre for HIV testing and care. In 2009, prior to our study, the annual report on malaria from this hospital indicated 4600 malaria cases that included 13 severe malaria-related deaths.

### 2.2 Study population

The target population of the study were febrile patients (with an axillary temperature of ≥37.5°C) who attended consultations or were hospitalised between January and August 2010. Patients of all ages that a) exhibited clinical signs that suggested malaria, b) who benefited from thick blood film (TBF) together with blood smear (BS) and c) gave oral consent to be screened for HIV infection and followed up according to our study schedule, were included. According to the Schlesselman formula [10], 30% is the proportion of patients actually suffering from malaria among a population of febrile patients in consultation for malaria; based on the results of a previous study of Menan *et al.* [5], with a 95% confidence interval (CI), an a-value of 5%, a b-value of 20%, a power (1−b) of 80% and a precision (i) of 5%, the number of patients to be included was at least 379 febrile patients, while using a rate of 10% patients lost to follow-up or withdrawal from the study.

We conducted a cross-sectional study amongst patients that had been treated for malaria during three consecutive days and followed these for a period of 28 days, according to the schedule Day0, Day3, Day7, Day14 and Day28. In the various study sites, with the assistance of the study teams, we collected the following information: Oral consent of the eligible patient, anamnestic data, clinical data, biological test results and drugs that resident physicians prescribed for treating malaria. The follow-up helped to assess treatment efficacy and safety, patient compliance and the outcome of all patients with TBF/BS retesting in case of fever at Day3, Day7, Day14, and Day28 but without polymerase chain reaction (PCR) correction.

Haematological and biochemical tests (blood glucose, creatininaemia, transaminases) and microscopy for TBF/BS were performed in the hospital laboratory. Uncomplicated malaria and severe malaria were defined by the presence of clinical signs and asexual forms of *P. falciparum* in the peripheral blood smear according to the 2003 WHO classification. In accordance with the current national guidelines [11], the treatment for uncomplicated malaria was artesunate + amodiaquine (ASAQ) as first-line therapy, then artemether + lumefantrine (AL) as alternative therapy. Quinine was dedicated for the treatment of uncomplicated malaria in pregnant women, for contraindication to ASAQ/AL in any patient and for severe malaria, with intramuscular (IM) artemether as alternative therapy. Intravenous artesunate was not yet recommended by the national guidelines at the time of the study.

### 2.3 Ethical considerations

The study was implemented in accordance with the national guidelines of the National Malaria Control Programme (NMCP) [11]. The antimalarials and materials used for microscopy were those provided to the public health centres. Only patients that provided oral consent were included. They were informed of the goal of the study, which was to improve medical care and disease diagnosis. Regular attendance and follow-up visits resulted in patients benefitting from free medical consultations and tests. Data confidentiality was guaranteed by assigning anonymous study numbers to patients in ascending and chronological order of inclusion, and by allowing only physicians in charge of patient follow-up to handle medical records.

### 2.4 Statistical analysis

Data were collected using a standard survey form and were analysed using Epi Info 6.0 software (CDC/OMS). The distribution of quantitative variables was described by the mean, standard deviation and interquartile range. Conversely, qualitative variables were described as total number and percentage. The Chi-square and Fisher’s exact tests, when appropriate, were used to compare proportions (with Yates’ correction). All tests were performed with a 5% significance threshold. The risk factors associated with malaria occurrence, severity and mortality were investigated using univariate analysis.

## 3 Results

**Figure 1.**
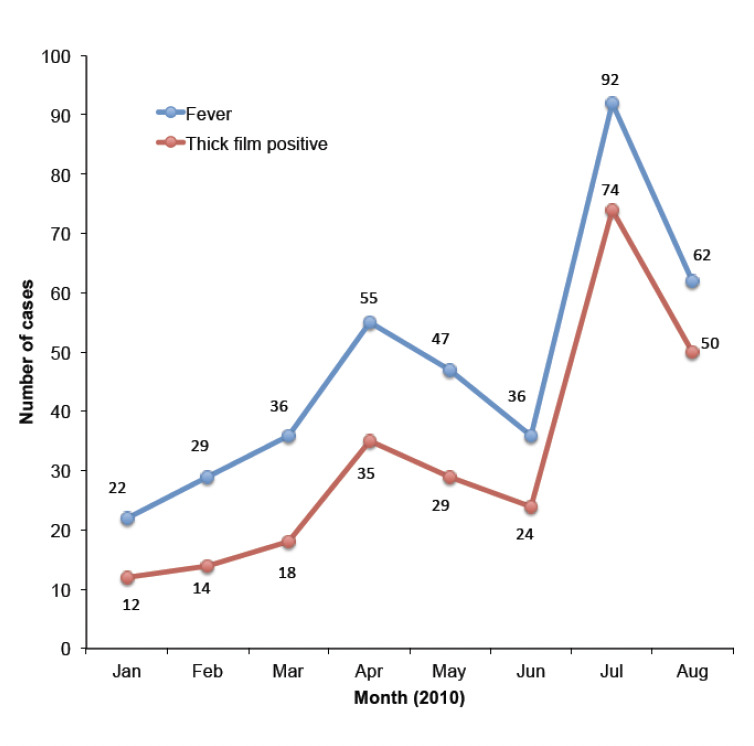
The monthly number of cases with fever and confirmed malaria in Tanda hospital, 2010.

Over the study period, 17,970 patients were seen by registered healthcare workers within the three study sites of GHT. Of these, 9106 patients (50.7%) were febrile. After performing an interview and physical examination of these febrile patients, 400 were suspected of having malaria and were therefore investigated for the presence of *Plasmodi-um* using TBF/BS. Of these 400 patients, 21 were excluded from the study after refusing to be included (3 cases), to undergo medical consultation (3), to provide blood samples (7), and for inability to read the slides due to electricity shortage (8). Out of 379 remaining patients, TBF was positive for 256 of these (67.5%). Both monthly incidence of confirmed malaria as well as fever episodes increased between January and August as well as fever episodes (Figure 1). Malaria prevalence was much higher in the rainy season (212/292 febrile patients in April-August) than in the dry season (44/87 febrile patients in January-March): 73% versus 51%, OR = 2.59; CI: 1.54-4.37; p<0.001.

The mean age of the 379 patients was 12±3.4 yrs and the median age was 4 yrs, with a range of one month to 71 yrs and a 51% proportion for patients between 6 months and 5 yrs of age. The majority of patients were living in an urban environment, particularly in Tanda (60%), where densely populated parts such as Zongo (30%) and Diou-labougou (15%) were housing more patients. Female patients accounted for 51.5% of cases, with 5 out of 13 pregnant women on intermittent preventive treatment with sul-fadoxine-pyrimethamine (IPT/SP; 4%).

About 40% of patients were using prevention methods promoted by the National Malaria Control Programme (long-lasting insecticide-treated nets (LLINs), indoor residual spraying (IRS), intermittent preventive treatment with IPT/SP, installation of mosquito-proof screens on windows and doors), whereas 26% were using less-recommended prevention methods (smoke, fan, traditional methods). The LLIN usage rate was 23%. Of these patients included, 33 were known to be HIV-1-infected (9%), of whom 16 were at the CDC clinical stages B and C (48.5%), 11 on cotrimoxazole prophylaxis for opportunistic infections (33%) and 18 on antiretroviral therapy (54.5%) over a mean period of 34±2.3 weeks (1-108 weeks) (Table 1).

**Table 1.** Sociodemogr aphic characteristics of the study population.

Parameter	Number (n=379)	%
**Gender**		
Male	184	48.5
Female	195	51.5
**Age**		
≤6 months	5	1.3
6 months -5 years]	192	50.7
5-15 years	85	22.4
15-60 years	93	24.5
>60 years	4	1.1
**Residence**		
Urban	231	60.9
Rural	148	39.1
**Occupation**		
Pupils/students	89	23.5
House keepers	57	15.0
Traders	24	6.3
Artists	15	4.0
Farmers	13	3.4
Workmen	12	3.2
Teachers	10	2.6
**Malaria prevention**		
LLIN	87	23
Fan	66	17.4
IRS	58	15.1
Smoke	29	7.5
IPT	5	1.3
Herbs	4	1
Screening	3	0.8
**HIV co-infection**	33	9
CD4 < 200,	6	1.6
CD4 [200-500]	10	2.6
CD4 > 500	6	1.6
Unknown CD4 count	11	2.9
Ongoing ART	18	4.7
Cotrimoxazole prophylaxis	11	2.9

### Malaria diagnosis

The armpit temperature ranged between 37.8°C and 43.0° C, with an average of 38.9°C. Headaches, asthenia and pain syndrome were reported in 38% of patients, whereas gastrointestinal symptoms were present in 150 patients (43%) and impaired consciousness in 23 patients (6.1%). The positivity rate of TBF was 67.5%. Only *P. falciparum* was identified, with a mean parasite density of 12,523±7,200 trophozoites/μl ranging between 100 and 86,000 trophozoites/μl of blood. This average density was 22,610 trophozoites/μl blood among HIV-infected patients and 10,055 trophozoites/μl blood among HIV-uninfected patients (p <0.001). A hyperparasitaemia > 20% was observed in 6 patients (Table 2). No gametocytaemia was detected. According to patient place of residence, the parasite rate was 71% in urban areas and 62% in rural areas (p = 0.18). The 256 confirmed malaria cases consisted of 59 uncomplicated malaria cases (23%) and 197 severe malaria cases (77%); thus, severe malaria was threefold more frequently observed than uncomplicated malaria. It was dominated by severe anaemia (83%), followed by cerebral malaria (10%) with convulsions, coma (Glasgow score < 9 among adults and Blantyre score ≥ 2 among children) than respiratory distress (4%) and hyperparasitaemia (3%). Malaria was more common in the rainy season (p < 0.001) among patients ≤ 5 years of age (p = 0.004) and among HIV-infected patients not receiving cotrimoxazole chemo-prophylaxis (p = 0.04). However, severe forms of malaria were more common during the rainy seasons (p < 0.001) and among patients ≤ 5 years of age (p < 0.001) (Table 3).

**Table 2.** Profile of patients with hyper parasitaemia (≥20%).

Parameter	Patient 1	Patient 2	Patient 3	Patient 4	Patient 5	Patient 6
Age (yrs)	6	2	3	3	3	1/3
Gender	F	M	F	M	F	F
Place of residence	Urban	Rural	Urban	Rural	Rural	Rural
HIV status	Negative	Negative	Negative	Negative	Negative	Negative
Major symptoms	Convulsion	Resp. dist.*	Convulsion	Resp. dist.	Resp. dist.	Convulsion
	Anaemic	Anaemic	Anaemic	Anaemic	Anaemic	Anaemic
Parasite density (µl)	60 000	86 000	53 000	65 000	65000	63 000
Hb level (g/dl)**	3,8	4,5	4,2	3,4	4,8	3,9
Malaria treatment	IM Art.***	IM Art.	IM Art.	IM Art.	IM Art.	IM Art.
Outcome	Cured	Death	Cured	Death	Cured	Death

* Resp. dist.: Respiratory distress; ** Hb: Haemoglobin; *** IM Art.: Artemether intramuscular injection.

**Table 3.** Factors associated with malaria occurrence and severity.

**Parameter**	**Malaria *n* = 256 (%)**	**No malaria *n* = 123 (%)**	**OR**	**95% CI**	***p*-value**
**Season**					
Rainy	212 (73)	80 (27)	2.59	1.54 – 4.37	0.0001
Dry	44 (51)	43 (49)			
**Age**					
≤ 5 years	146 (74)	51 (26)	1.87	1.18 – 2.97	0.004
> 5 years	110 (60)	72 (40)			
**HIV (+) patients**					
Cotrimoxazole	2 (18)	9 (82)	0.15	0.02 – 0.96	0.04
no cotrimoxazole	18 (60)	12 (40)			
**Parameter**	**Severe malaria *n* = 197 (%)**	**Uncomplicated malaria *n* = 59 (%)**	**OR**	**95% CI**	***p*-value**
**Season**					
Rainy	174 (82)	38 (18)	4.18	1.99 – 8.80	0.00002
Dry	23 (52)	21 (48)			
**Age**					
≤ 5 years	125 (86)	21 (14)	3.14	1.64 – 6.03	0.0001
> 5 years	72 (65)	38 (35)			

### Therapeutics and outcome of patients

IM artemether was more frequently prescribed (56.3%) than quinine (28.1%), LA (9.8%) and ASAQ (6%). In uncomplicated malaria, ASAQ was twice less prescribed than AL, while in severe malaria quinine was three times less prescribed than IM artemether (Table 4). During follow-up, apyrexia and parasite clearance were observed at D2 and D3 in 223 patients (87%). Two recurrence cases were observed at D23 and D25. They were successfully retreated with the same drug taken in the first episode (AL). Adverse events (AEs) attributable to antimalarial drugs were reported in 44 clinical records (17%): 16 cases of tinnitus, 14 cases of pruritus, 10 cases of nausea, 9 cases of asthenia, 7 cases of abdominal pain, 4 cases of dizziness, making a total of 60 events reported among 44 patients (Table 4).

**Table 4.** Patient outcome as a result of various malaria treatments.

Treatment	Treatment *n* = 256 (%)	AE* *n* = 60 (%)	Lost during follow-up *n* = 13 (%)	Cured *n* = 230 (%)	Died *n* = 13 (%)
IM Artemether	144 (56.3)	2 (3.3)	0 (0)	134 (58.3)	10 (77)
IVor Oral Quinine	72 (28.1)	30 (50)	0 (0)	69 (30)	3 (23)
Oral AL	25 (9.8)	18 (30)	9 (69.2)	16 (7)	0 (0)
Oral ASAQ	15 (5.9)	10 (16.7)	4 (21.8)	11 (4.8)	0 (0)

* AE: Adverse Event; IM: Intramuscular; IV: Intravenous; AL: Artemether + Lumefantrine; ASAQ: Artesunate + amodiaquine.

The evolution of patients was marked by 230 cases of clinical healing (90%), 13 patients lost to follow-up (5%) and 13 deaths (5%). Death occurred in 197 patients with severe malaria, with a specific lethality of 7% for the severe form of malaria. This lethality was statistically associated with age ≤ 5 years (p = 0.02), the presence of neurological disorders (p < 0.001), respiratory distress (p = 0.007) and hyperparasitaemia (p = 0.004) (Table 5).

**Table 5.** Factors associated with the lethality of severe malaria.

Factor	Died *n* = 13	Cured *n* = 184	OR	95% CI	*p*-value
**Age**					
≤ 5 years	12	113	7.54	0.98-158.5	0.02
> 5 years	1	71			
**Neurologic disorders**					
Present	8	12	22.9	5.63-98.32	<0.00001
Absent	5	172			
**Respiratory distress**					
Present	3	4	13.5	2.04-87.5	0.007
Absent	10	180			
**Hyperparasitaemia**					
Present	3	3	18	2.49-135	0.004
Absent	10	181			

## 4 Discussion

Developed to cover one full year, the study could only be carried out over a period of 8 months (January-August 2010) because of the military-political turmoil experienced in Côte d’Ivoire during the election period around October 2010, which poses a limitation to our study. However, it has the merit of including two rainy seasons, in which we observed an increase in malaria cases in Ivory Coast. Thus, the proportions of confirmed cases will serve as records for an area where malaria cases had been reported presumptively.

The majority of the study population originated from the various areas of Tanda city and only rarely from rural areas. This confirms the current malaria emergence in urban areas, which is a phenomenon related to several factors, namely the growing and uncontrolled urbanisation, urban and peri-urban agriculture, irrigation, the expansion of drug resistance, and especially entomological and climatic changes observed in recent years [12-14]. These factors may explain heterogeneous transmission in urban areas due to the decrease in the prevalence of hematozoa carriers and especially the loss of immunity in many urban subjects. Thus, in major African cities, the parasite rate is higher in less sanitized suburbs than in central districts where vectors are scarce and access to medical care easier [15]. This result confirms that malaria risk is still widespread in African endemic areas where few people actually use effective preventive methods. Consequently, in spite of the numerous high-profile mosquito net distribution campaigns to protect vulnerable subjects in priority, children remain the primary target of malaria since 74% of our study sample consisted of children aged below 15 yrs and children below the age of 5 yrs accounted for 86% of severe malaria cases as revealed by other studies [16,17].

Given our results, the percentage of HIV-infected subjects is as low as that recorded in a multicentre study conducted in Dakar, Bamako, Bobodioulasso and Abidjan [18]. However, it is 9% lower than published in other African studies [19-21]. The high prevalence of HIV-1 infection previously reported by Soumaré *et al.* in Dakar [21] and Eholié *et al.* in Abidjan [20] confirms the strong presence of this viral type throughout the region. However, our results draw people’s attention to the need to promote the management of HIV-infected patients in northern Côte d’Ivoire where less than half of patients had simultaneously received cotrimoxazole prophylaxis for opportunistic infections and antiretroviral therapy despite the late stage of their disease, whereas early treatment is currently recommended [22].

The clinical pictures observed in our study are similar to those described elsewhere in Africa [23]. In uncomplicated malaria, fever is the main symptom, frequently associated with pain syndrome and asthenia. On the other hand, apart from fever, severe malaria is marked by respiratory distress, severe anaemia and neurological disorders, including coma and convulsions, which are usually the most symptoms [24]. However, compared with previous findings, the particularity of our work lies in two major factors: a significant hyperparasitaemia in 2% of patients and a marked predominance of severe malaria (77%) which was threefold more commonly observed than uncomplicated malaria (23%). These facts may be explained by the high proportion of subjects with no premunition (young children or HIV-infected subjects), the importance of massive infestation during rainy seasons and a general hospital considered as a last resort for patients in critical condition coming from the first contact clinics of the sanitary district. Thus, this predominance of severe forms of malaria was notified in 2006 by Soumaré *et al.* with a 66% frequency in Dakar [21]. However, Eholié *et al.* [20] reported in 2004 in Abidjan a proportion of 17%. These results show that the risk of severe malaria in increases from large urban agglomerations to secondary or peripheral cities where conditions exposing humans to transmission are more prevalent [15].

The overall prevalence of malaria was 67.5% in febrile patients investigated with TBF in a referral hospital. Our study thus highlights a significant difference between the incidence of confirmed malaria and the presumptive diagnosis of malaria based solely on the presence of fever. Indeed, at the same site, the 2009 annual report of the sanitary district reported 14,856 clinical malaria cases, including 4,600 cases at the general hospital of Tanda, whereas in 2010, we only confirmed 256 cases among 379 cases of presumptive malaria. Although our study was conducted for only 8 months, this difference results from the usual overestimation of malaria incidence without parasitological confirmation. This shows the importance of biological diagnosis as a major tool for monitoring a malaria control programme, inasmuch as this alone can provide information on the fluctuation in malaria incidence. Malaria incidence varied, with a predominance in the densely populated and poor neighbourhoods of Tanda city, such as Zongo (30%) and Dioulabougou (15%). However, the *P. falciparum parasite rate* of 62% observed in rural areas is superimposable on that reported in 1994 by Dossou *et al.* [25] in Allokokro, a village located in the wet savannah. On the other hand, it is lower than the 85% rate observed in the Tai forest area by Nzeyimana *et al.* [26]. We can conclude that, regardless of the population age structure, the risk of malaria parasite transmission varies between sites, which is a phenomenon already observed in Brazzaville, where we have noticed transmission to vary strongly from one area to another, ranging from less than one infectious bite per person every two years to more than hundred infectious bites per year [15].

Our results show that malaria prevalence was high at the GHT among presumptive cases. However, it was significantly related to the rainy season, children’s young age and HIV infection. These data confirm the results by Ndiaye *et al.* [27] and Eholié *et al.* [20], which identified the rainy season as a factor for malaria over-morbidity in Dakar and Abidjan. Unlike in the forested area of Tai, where malaria prevalence is stable all year long [26], the rainy season, the young age of children under 5 yrs, the deficit in CD4 T-cells among HIV-infected patients and the non-use of antimalarial prevention measures were also factors associated with malaria. However, despite the reduced number of HIV-infected patients, the study confirms the protective efficacy of cotrimoxazole against the risk of malaria [28,29].

The study also generates interest in the choice of treatment regimens and patient outcomes. Indeed, until 2012, the national guidelines recommended ASAQ combination as first-line treatment, then AL combination as alternative treatment for uncomplicated malaria. However, in our study, despite these guidelines, ASAQ combination was half as frequently prescribed as AL combination. Moreover, given the high frequency of severe anaemia and the cost of IV infusion therapy, IM artemether was prescribed three times more often than IV quinine for severe malaria. Nevertheless, all drugs were equally effective and switching between treatments did not affect treatment safety or efficacy, marked by a 5% mortality rate, which is comparable to that reported from other African studies [20,21]. However, our results also inspired the NMCP to revisit the national guidelines by now recommending AL or ASAQ as first-line therapy for uncomplicated malaria and IV artesunate for severe malaria.

Considering only severe malaria, its lethality rate in our study remains lower (7%) than that reported by Niyongabo *et al.* [19] and Eholié *et al.* [20], namely 23% in Bujumbura and 15.4% in Abidjan. However, despite the antiplasmodial efficacy of drugs, the prognosis for cases of severe malaria is still bleak. An age under 5 yrs, respiratory distress, hyperparasitaemia, neurological disorders and particularly the absence of intensive care units are major factors for poor prognosis [20]. In Malaysia, Koh *et al.* [30] reported in 2004 a higher lethality rate of 62.5% associated with acute renal failure, respiratory distress, disseminated intravascular coagulation and acute liver failure. In Abidjan, Kouamé *et al.* incriminated the same serious complications but the lethality rate was lower thanks to appropriate intensive care units that are more often lacking in peripheral hospitals in Africa [31].

## 5 Conclusions

This study revealed that at the general hospital of Tanda, children <5 yrs remain the main victims of malaria, with a clear predominance of severe malaria in this age cohort. Morbidity is more prominently associated with the rainy season, young age and HIV infection. Among HIV-infected patients, cotrimoxazole used in prophylaxis for opportunistic infections has a protective effect against malaria. This work emphasises the interest in strengthening prevention measures against malaria in this district especially during rainy seasons, but also measures for the treatment of severe forms of malaria. In view of these observations it is advisable to better equip the general hospital of Tanda to manage severe and complicated malaria.

## References

[2] Ndiaye P, Tal-DIA A, Diedhiou A, Juergens-Behr A (2006). L’automédication de la fièvre dans le district nord de Dakar, au Sénégal.. Méd. Trop..

[3] Baudon D, Gazin P, Galaup B, Pellotier-Guinart E (1988). Fiabilité de l’examen clinique dans le diagnostic des fièvres palustres en zone d’endémie ouest-africaine.. Méd. Trop..

[4] Reyburn H, Mbatia R, Drakeley C, Carneiro I (2004). Over-diagnosis of malaria in patients with severe febrile illness in Tanzania: a prospective study.. BMJ.

[5] Menan EIH, Yavo W, Oga SSA, Kiki-Barro PC (2007). Diagnostic clinique présomptif du paludisme: part réelle de la maladie.. Méd Afr. Noire.

[6] D'Acremont V, Kilowoko M, Kyungu E, Philipina S (2014). Beyond malaria - causes of fever in outpatient Tanzanian children.. N. Engl. J. Med..

[7] World Health Organization: Assessment and monitoring of antimalarial drug efficacy for the treatment of uncomplicated falciparum malaria. (2003).

[8] Aba YT, Bissagnené E, Kra O, Assi SB (2014). Performance and clinical usefulness of the Optimal-IT® test in the treatment of confirmed malaria cases in rural areas in Côte d’Ivoire.. MalariaWorld J..

[10] Schlesselman JJ (1974). Sample size requirements in cohort and case control studies of diseases.. Am. J. Epi..

[11] Programme National de Lutte contre le Paludisme, Minis-tère de la Santé et de l’Hygiène Publique, Côte d’Ivoire. (2008). Directives nationales de prise en charge du paludisme en Côte d’Ivoire, version révisée février.

[12] Trape JF, Zoulani A (1987). Malaria and urbanization in Central Africa: The example of Brazzaville. Relationship between urbanization and the intensity of transmission.. Trans. R. Soc. Trop. Med. Hyg..

[13] Fondjo E, Robert V, Le Goff G, Toto JC (1992). Le paludisme urbain à Yaoundé (Cameroun): étude entomologique dans 2 quartiers périurbains.. Bull. Soc. Pathol. Exot..

[14] Akogbeto M (2000). Le paludisme côtier lagunaire à Cotonou : données entomologiques.. Cahiers Santé.

[15] Carme B, Hayette MP, Mbits A, Samba G (1995). Indice plasmodique et parasitémie pronostic au Congo.. Ann. Soc. Belge Méd. Trop..

[16] Tietche F, Teguia S, Tetanye E, Louis F (1996). Diagnostic présomptif d’accès palustre et positivité de la goutte épaisse chez l’enfant de 0 à 5 ans à Yaoundé (Cameroun).. Méd Afr. Noire..

[17] Assoumou A, Adoubryn KD, Aboum KS, Kouadio-Yapo CG (2008). Portage symptomatique et asymptomatique de Plasmodium falciparum chez les enfants de 6 mois à 6 ans à l’hôpital général d’Abobo (Abidjan, Côte d’Ivoire).. Bull. Soc. Pathol. Exot..

[18] Ello NF, Eholié S, Cissé H, Sawadogo A

[19] Niyongabo T, Deloron P, Aubry P, Ndarugirire F (1994). Prognostic factors in adult cerebral malaria: a case study in Burundi, an area on high prevalence of HIV infection.. Acta Trop..

[20] Eholié SP, Ehui E, Adou-Bryn K, Kouame KE (2004). Paludisme grave de l’adulte autochtone à Abidjan (Côte d’Ivoire).. Bull. Soc. Pathol. Exot..

[21] Soumaré M, Seydi M, Diop SA (2008). Place du paludisme dans un service de pathologie infectieuse à Dakar (Sénégal).. Méd Trop..

[23] Diallo AH, Guiguemdé TR, Ki-Zerbo G (2003). Aspects cliniques et parasitologiques du paludisme grave de l’adulte en milieu urbain de Bobo-Dioulasso (Burkina-Faso).. Bull. Soc. Pathol. Exot..

[24] Snow RW, Guerra CA, Noor AM, Myint HY (2005). The global distribution of clinical episodes of Plasmodium fal-ciparum malaria.. Nature.

[25] Dossou JY, Ouattara A, Doannio JMC, Rivière F (1994). Aspects du paludisme dans un village de savane humide de Côte d'ivoire.. Med. Trop..

[26] Nzeyimana I, Henry MC, Dossou JY, Doannio JMC (2002). Epidémiologie du paludisme dans le sud-ouest forestier de la Côte d’Ivoire (région de Taï).. Bull. Soc. Pathol. Exot..

[27] Ndiaye O, Le Hesran JY, Etard J F, Diallo A (2001). Variations climatiques et mortalité attribuée au paludisme dans la zone de Niakhar, Sénégal, 1984 à 1996.. Cahiers Santé.

[28] Mermin J, Lule J, Ekwaru JP, Malamba S (2004). Effect of cotrimoxazole prophylaxis on morbidity, mortality, CD4-cell count, and viral load in HIV infection in rural Uganda.. Lancet.

[29] Mermin J, Lule J, Ekwaru JP, Downing R (2005). Cotrimox-azole prophylaxis by HIV-infected persons in Uganda reduces morbidity and mortality among HIV-uninfected family members.. AIDS.

[30] Koh KH, Chew PH, Kiyu AA (2004). Retrospective study of malaria infections in an intensive care unit of a general hospital in Malaysia.. Singapore Med. J..

[31] Kouamé K, Brouh Y, Soro L, Bissagnené E (2002). Palu-disme grave chez les expatriés en réanimation à Abidjan.. Ann. Fr. Réanim..

